# Abnormal motor activity during anaesthesia in a dog: a case report

**DOI:** 10.1186/1751-0147-52-64

**Published:** 2010-12-01

**Authors:** Andreas Lervik, Henning A Haga, Max Becker

**Affiliations:** 1 Department of Companion Animal Clinical Sciences, Norwegian School of Veterinary Science, Oslo, Norway

## Abstract

Seizures or convulsions that occur during anaesthesia in veterinary patients are infrequently reported in the literature. Consequently, the incidence of such events is unknown. Several drugs commonly used in clinical veterinary anaesthesia have been shown to induce epileptiform activity in both human clinical patients and experimental candidates. The present case report describes convulsions in a four-year old male Bernese mountain dog during maintenance of anaesthesia with isoflurane after premedication with acepromazine and methadone followed by co-induction with propofol and ketamine. The dog had no history of previous convulsions. The use of several sedative and anaesthetic drugs makes it difficult to find one single causative pharmaceutical.

## Background

The classification of abnormal motor activity in veterinary patients is unclear. The international league against epilepsy (ILAE) guidelines have been used, with various modifications, as a basis for classification of seizure types in dogs [1] An epileptic seizure involves, according to ILEA a clinically manifested event with a corroborative EEG abnormality, and the clinical observed state is described as a convulsion [2]. A genetic background for canine epilepsy has been described in several breeds including the Bernese Mountain Dog [1,3]. Seizures or convulsions in conjunction with clinical veterinary anaesthesia are infrequently reported in the literature, and the incidence of such events or their relation to genetic susceptibility for epilepsy is not known. Several drugs commonly applied in clinical veterinary anaesthesia may induce epileptiform activity in human clinical patients and in experimental studies [2]. Multimodal analgesia and inducing anaesthesia with more than one drug have become increasingly popular over the last years, leading to the use of a wide range of drug combinations in small animal anaesthesia [4-6]. This case report describes convulsions in a dog during isoflurane anaesthesia after premedication with acepromazine and methadone followed by co-induction with propofol and ketamine.

## Case presentation

A four years old male Bernese mountain dog weighing 45 kg underwent general anaesthesia for arthroscopy of the left stifle joint and a tibia plateau levelling osteotomy of the same leg. The dog had no previous history of disease, and a preanaesthetic physical examination revealed no abnormalities except from a ruptured cranial cruciate ligament on the left hind leg. Ten days earlier the dog had been sedated for a radiological examination of the left stifle joint with 0.36 mg/kg of xylazine given intravenously without any noticeable adverse effects. On the day of surgery, the dog was premedicated with an intramuscular injection of 50 μg/kg acepromazine (Plegicil Vet 10 mg/ml, Pharmaxin, Helsingborg, Sweden) and methadone 0.1 mg/kg (Metadon 10 mg/ml, Sykehusapoteket Rikshospitalet, Oslo, Norway). An 18 gauge intravenous catheter (Becton Dickinson, Helsingborg, Sweden) was placed in the left cephalic vein. General anaesthesia was induced by an intravenous injection of ketamine 2 mg/kg (Ketalar 50 mg/ml, Pfizer Inc, New York, USA) immediately followed by an intravenous injection of propofol (Propofol-Lipuro 10 mg/ml, B. Braun, Melsungen, Germany) given through the intravenous catheter to facilitate endotracheal intubation. The dog was placed in right lateral recumbency and allowed to breathe spontaneously. Anaesthesia was maintained with isoflurane (Isoba Vet., Schering Plough, Ballerup Denmark) in an equal mixture of oxygen and air delivered by means of a circle patient breathing system connected to an anaesthetic machine (Matrx. Medical Inc., Orchard Park, NY, USA). The dog received an infusion of Ringers acetate (Ringer Acetate, Fresenius Kabi, Halden Norway) at 10 ml/kg/hour. A multi-parameter-anaesthetic monitor (Datex Ohmeda S/5 compact anesthesia monitor, Datex-Ohmeda Instrumentarium corp., Finland) was used to continuously monitor systolic, diastolic and mean arterial blood pressure, a 3-lead electrocardiogram, SpO_2_, FiO_2_, E_T_CO_2_, E_T _isoflurane (E_T_iso) and rectal temperature.

Approximately 10 minutes after induction of anaesthesia the dog displayed clonic muscular activity of the front limbs and head, including paddling of the front limbs and twitching of lips and eye lids. Concurrently heart rate and respiratory rate increased from 102/min to 126/min and 17/min to 47/min respectively. E_T_iso concentration at this point was 1.0 Vol%. Midazolam (Midazolam 5 mg/ml, B. Braun, Melsungen, Germany) 0.1 mg/kg was administered intravenously and the inspired isoflurane fraction (F_i_iso) was increased. This resulted in cessation of the convulsions. An arterial blood sample was simultaneously collected and analysed using an automated blood gas analyser (ABL 800 Flex, Radiometer Copenhagen, Brønshøj, Denmark). The blood gas analysis revealed a respiratory alkalosis with a pH of 7.533, a PaCO_2 _of 2.64 kPa and a base excess of - 4.0 mmol/l, but no abnormalities in PaO_2_, ionised calcium, sodium, potassium, chloride, lactate, glucose or haemoglobin concentrations.

Convulsive activity as previously described resumed again after about 10 minutes from the first occurrence. E_T_iso was now 1.5 Vol%. Heart rate again increased from 100/min to 125/min and the respiratory rate increased to 59/min after a period of apnoea during which the dog was ventilated manually. At this point, body temperature measured rectally was 35.6°C. E_T_iso was decreased to1.1 Vol%. Midazolam 0.1 mg/kg was administered i.v. without apparent effect upon the convulsions. Thiopental (Pentothal Natrium 500 mg, Hospira Ent., Hoofddorp, Netherlands) 1 mg/kg i.v was administered twice. The convulsions continued, and phenobarbital (Fenobarbital natrium NAF 100 mg/ml, NAF, Oslo, Norway) 4 mg/kg was given i.v. followed by a third dose of thiopental at 1 mg/kg. At this point the convulsions subsided 45 minutes after the first convulsions started. Isoflurane delivery was discontinued and the dog was allowed to recover. Thirty minutes after discontinuing isoflurane delivery the dog had had no further convulsions, and was lying in sternal recumbency.

The following day a neurological examination was performed and a blood sample was also analysed, including a complete blood count and a full biochemical evaluation, without revealing any abnormalities. Two days after the first anaesthesia it was decided to anaesthetise the dog a second time to perform the procedure not undertaken due to the described adverse event. An intravenous catheter was placed and general anaesthesia was induced by injection of 0.4 mg/kg of midazolam combined with fentanyl (Fentanyl Hameln 50 μg/ml, Hameln Pharmaceuticals, Hameln, Germany) until intubation was possible. The dog was endotracheally intubated, and anaesthesia was maintained by an infusion of fentanyl at 20-40 μg/kg/h and midazolam at 0.2-0.3 mg/kg/h. The dogs ventilation was controlled using an automatic ventilator throughout the anaesthesia to maintain normocapnia. A lumbosacral epidural injection of 0.2 ml/kg 0.5% bupivacaine (Marcain 5 mg/ml, AstraZeneca, Södertälje, Sweden) was performed. After starting surgery sevoflurane (Sevoflo, Abbott lab, Kent, Great Britain) was added to the mixture of oxygen and air to achieve end tidal sevoflurane concentration of 0.9 - 1.5 Vol%. No convulsive activity was observed and the dog recovered uneventfully from anaesthesia. Ten months after the incidence during anaesthesia the dog was behaving normal according to the owner, no further sedative or anaesthetic drug has been given the dog, and no further convulsions or seizures had been observed.

## Discussion

This case report describes myoclonic convulsive activity in a dog following administration of several sedative and anaesthetic drugs making it difficult to point out one single causative factor. Acepromazine has been cited in the literature to cause a lowering of seizure threshold in dogs, and it has been stated that it should be avoided in dogs with a history of epilepsy or convulsions [7]. Aliphatic phenothiazines such as chlorpromazine have been shown to reduce seizure threshold in dogs and humans. In dogs with a history of seizures without known underlying disease, a rapid intravenous injection of 2.2 mg/kg chlorpromazine potentiated or induced EEG changes in 22 of 43 dogs and precipitated seizures in two of the dogs [8]. In a study from 1967, a higher incidence of seizures was found in psychiatric patients without a history of epilepsy treated with chlorpromazine (25 to over 1000 mg/day) than the incidence of a first unprovoked seizure in the general population (0.07-0.09%) [9]. The incidence of seizures following the use of phenothiazines is unknown in canine patients. In a recent study, 27 of 31 dogs with a known history of seizures did not display seizure activity in an observational period after treatment with acepromazine. The majority of these dogs had however received antiepileptic medications prior to treatment [10]. In a second retrospective study, acepromazine did not increase the risk of seizures in dogs with a history of seizures due to different aetiologies, and was also used to treat seizure activity successfully [11]. The dose of acepromazine administered in the current case and in the studies describing use in dogs with a history of seizures are more than a tenfold lower than the dose of chlorpromazine cited to cause EEG changes in dogs, while the route of administration varies between the different cases described. This could account for some of the discrepancy between the reports. Whether acepromazine contributed to the development of seizures or not in the current case remains unknown.

NMDA receptor blockade should theoretically be able to ameliorate epileptiform activity [12] and as such, ketamine has been used to treat refractory *status epilepticus *in humans and a dog [13]. Ketamine at higher doses has on the other hand been associated with convulsions, increased muscle tone and spontaneous muscular activity in dogs [14]. It has also been advised that ketamine should not be used in animals with pre-existing seizure disorders [15]. Although the evidence is conflicting, ketamine is probably a better anticonvulsant than a proconvulsant when used with a GABA-agonist [2,16]. The time elapsed from injection of ketamine to the onset of the abnormal muscle activity was about 10 to 12 minutes in the described case; this corresponds with the time span where maximal motor side effects are described to present in dogs when ketamine is given alone [14]. In a previous study using lower doses of ketamine for co-induction of anaesthesia with propofol, none of the dogs displayed convulsions or seizures after ketamine administration. The examiners did not find a difference in motor side effects in dogs administered the combination of ketamine and propofol compared to when propofol was used alone for induction of anaesthesia [4]. In human patients the use of ketamine combined with propofol for induction of anaesthesia reduced the incidence of excitatory effects significantly in comparison to use of propofol alone [17]. The fact that the dose of ketamine chosen in the present case was relatively low and that it was used for co-induction of anaesthesia with the GABA agonist propofol after premedication with acepromazine makes it less likely that ketamine was responsible for the convulsions observed.

A number of neurological complications including abnormal motor activity have been associated with the use of propofol for induction and maintenance of general anaesthesia in human patients [18]. Abnormal motor activity in conjunction with the use of propofol for induction and maintenance of anaesthesia has also been described in dogs, with a reported incidence between 7.5 to 25%, and with clinical signs such as tremor, tics, muscular rigidity of the forelimbs, swimming movements, opisthotonus and nystagmus most common [19-21]. Paradoxically, propofol has depressant effects on the central nervous system involving GABA, glutamate and aspartate mechanisms [22], and has been used successfully as an anticonvulsant in human and veterinary patients with seizures [23,24]. Although the onset of the clinical signs was delayed, propofol could be responsible for the signs displayed by the dog in this case report. A late onset of clinical signs up to 30 minutes after the end of propofol anaesthesia has been described in human patients [18], and propofol has a prolonged terminal elimination half life in dogs and humans, leading to significant concentrations of propofol in the nervous system for some time after injection [25,26].

Volatile anaesthetics have been associated with induction of abnormal EEG changes, seizures or abnormal muscular activity in humans and other species, although a difference seems to be present between agents regarding their pro- and anticonvulsant potentials [2,2,27]. Enflurane is known to produce EEG changes and clinical signs of abnormal muscular activity in dogs, while sevoflurane was not found to induce seizure activity [28]. The epileptogenic effect of sevoflurane, although controversial, has been frequently reported in humans. The effect might be dose related and can be precipitated by hypocapnia [29]. In the described case, a peri-MAC concentration of isoflurane was used to maintain anaesthesia as abnormal muscular activity occurred. The incidence of seizures during isoflurane anaesthesia is controversial [30], but seizures during recovery have been reported in humans [31]. The nonconvulsant effects of isoflurane, seem, however, to be more important [2], and isoflurane has been used successfully in the treatment of *status epilepticus *in human patients [32]. The role of isoflurane in the current case is not known, but a contribution to the development of convulsions can not be excluded.

The dog described in this case report had no previous history of convulsions, neither were signs of disease found during the pre-anaesthetic examination nor after a neurological examination and blood analysis. A predisposition of idiopathic epilepsy seems to be present in the Bernese mountain dog [3], and underlying disease can not be excluded as a contributing cause of the observed clinical signs.

Diazepam, phenobarbital and pentobarbital have all been suggested for the treatment if severe signs develop in conjunction with the use of propofol in dogs [20,25]. This is a similar approach to the one applied to control clinical signs in the present case, where benzodiazepines and barbiturates combined with discontinuation of anaesthesia seemed to alleviate signs of abnormal muscular activity.

The choice of protocol for the second anaesthesia in the present dog was based on exclusion of the drugs used during the first anaesthesia, and the usage of drugs known to have little potential of causing abnormal muscular activity in dogs, humans and other species. Midazolam is a benzodiazepine, that acts mainly on the α GABA_A _subunit and is known to be strongly anticonvulsant [2]. On the other hand, dogs that receive midazolam alone may show signs of agitation or increased excitement [5,33]. Mu-agonistic opioids can cause seizures in human patients [2], but at the same time fentanyl has been shown to reduce the occurrence of epileptiform spike waves during sevoflurane anaesthesia in humans [34]. An end, tidal concentration of sevoflurane well below the published MAC for dogs was used to maintain anaesthesia in the current case [35]. This corresponds to recommendations made for the use of sevoflurane in human patients to minimise the epileptogenic potential of sevoflurane [29]. The anaesthetic regime used for this second anaesthesia seemed to be successful in preventing development of abnormal muscular activity. However, EEG monitoring was not performed and epileptiform activity could have been present without clinical signs. Phenobarbital has a long terminal half life in dogs [36], and a persisting anticonvulsant effect of the injection given 2 days earlier can not be entirely excluded.

## Conclusions

This case report describes convulsions in a dog during anaesthesia achieved by use of multiple drugs for balanced anaesthesia used to minimise side effects. Treatment with benzodiazepines and barbiturates combined with discontinuation of anaesthesia could successfully be used to stop convulsions, and a second anaesthetic procedure was successfully performed by excluding all drugs used in the first anaesthetic procedure and choosing drugs known to have a low potential of causing convulsions in humans and dogs. The use of multiple drugs during anaesthesia has several advantages, but can also make it difficult to point out a single causative pharmaceutical if unwanted effects occur.

## Consent

Written informed consent was obtained from the owners for publication of this case report. A copy of the written consent is available for review by the Editor-in-Chief of this journal.

## Competing interests

The authors declare that they have no competing interests.

## Authors' contributions

AL planned and performed anaesthesia and perioperative care in this patient and is the main author of the paper. MB supervised the anaesthesia and perioperative care in this patient. HAH contributed to the second anaesthesia in this patient. All authors read and approved the final manuscript.
